# Decoding Semantics from Dynamic Brain Activation Patterns: From Trials to Task in EEG/MEG Source Space

**DOI:** 10.1523/ENEURO.0277-23.2023

**Published:** 2024-03-01

**Authors:** Federica Magnabosco, Olaf Hauk

**Affiliations:** MRC Cognition and Brain Sciences Unit, University of Cambridge, 15 Chaucer Road, Cambridge CB2 7EF, United Kingdom

**Keywords:** decoding, MVPA, semantic cognition, semantics, task, visual word recognition

## Abstract

The temporal dynamics within the semantic brain network and its dependence on stimulus and task parameters are still not well understood. Here, we addressed this by decoding task as well as stimulus information from source-estimated EEG/MEG human data. We presented the same visual word stimuli in a lexical decision (LD) and three semantic decision (SD) tasks. The meanings of the presented words varied across five semantic categories. Source space decoding was applied over time in five ROIs in the left hemisphere (anterior and posterior temporal lobe, inferior frontal gyrus, primary visual areas, and angular gyrus) and one in the right hemisphere (anterior temporal lobe). Task decoding produced sustained significant effects in all ROIs from 50 to 100 ms, both when categorizing tasks with different semantic demands (LD-SD) as well as for similar semantic tasks (SD-SD). In contrast, a semantic word category could only be decoded in lATL, rATL, PTC, and IFG, between 250 and 500 ms. Furthermore, we compared two approaches to source space decoding: conventional ROI-by-ROI decoding and combined-ROI decoding with back-projected activation patterns. The former produced more reliable results for word category decoding while the latter was more informative for task decoding. This indicates that task effects are distributed across the whole semantic network while stimulus effects are more focal. Our results demonstrate that the semantic network is widely distributed but that bilateral anterior temporal lobes together with control regions are particularly relevant for the processing of semantic information.

## Significance Statement

Most previous decoding analyses of EEG/MEG data have focussed on decoding performance over time in sensor space. Here, for the first time, we compared two approaches to source space decoding in order to reveal the spatiotemporal dynamics of both task and stimulus features in the semantic brain network. Our results inform the spatio-temporal dynamics of semantic word processing, with task information being decodable already at early latencies in multiple brain regions, while stimulus information becomes available later and more focally. Our two decoding approaches performed differently for these effects, with ROI-by-ROI decoding more sensitive to focal and combined-ROI decoding more sensitive to distributed effects. Our results suggest that single-word semantic information is at least partially stable across tasks.

## Introduction

Semantic cognition is the representation and processing of the acquired knowledge about the world. The semantic brain network that enables us to store, employ, manipulate, and generalize conceptual knowledge can be characterized along two dimensions: representation and control (Lambon Ralph et al., 2017). Bilateral anterior temporal lobes (ATLs) constitute a multimodal hub within the representation component, where modality-specific information is combined to represent coherent concepts (Patterson et al., 2007). However, context and task influence the type of information that is relevant in any particular moment, requiring a control system capable of manipulating and shaping the activations in the representation system (Jefferies, 2013). A recent meta-analysis indicated that the control system is mostly left-hemispheric and comprises the inferior frontal gyrus (IFG) and posterior temporal cortex (PTC; Jackson, 2021). However, little is known about the dynamics within and between the components of the semantic network. In this study, we are tapping into semantic brain dynamics by decoding tasks as well as stimulus features from source-estimated EEG/MEG data in a visual word recognition paradigm.

In neuroimaging research, EEG/MEG and fMRI datasets have traditionally been analyzed with different decoding/multivoxel pattern analysis approaches (Grootswagers et al., 2017). Due to its limited temporal resolution, fMRI is commonly used to estimate brain areas [e.g., regions of interest (ROIs) or local spheres] where stimulus and task features can successfully be decoded (Haxby et al., 2001; Huth et al., 2016). In contrast, because of EEG/MEG's limited spatial resolution, most previous studies have focussed on the time course of decoding accuracy obtained in sensor space (Cichy et al., 2014; Gwilliams et al., 2023). However, ROI-wise decoding in source space is possible using appropriate distributed source estimation methods (Kietzmann et al., 2019), generating information about both temporal and spatial aspects. Alternatively, it has been proposed to interpret the topography of classifier weights from the decoding analysis and, for example, submit them to source estimation. In this case, the weights have to be back-projected into activation patterns before the application of source estimation (Haufe et al., 2014). This back-projection method should be applied whenever the classifier patterns themselves (rather than decoding accuracy) are interpreted, since otherwise seemingly high contributions from some sensors or voxels may be due to noise rather than signal (Haufe et al., 2014). This offers another approach to decoding analysis in source space: One can apply decoding to data across all ROIs that are assumed to be involved in the processes of interest and interpret the distribution of these decoding weights across ROIs following back-projection, thus determining which regions contribute more or less to the successful decoding of stimulus or task information. To our knowledge, these two approaches—conventional ROI-by-ROI decoding and across-ROI decoding with back-projection—have not been applied to the same EEG/MEG dataset in source space yet. The ATLs are sensitive to the semantic characteristics of single words as shown in several EEG/MEG studies both in univariate analyses (Marinkovic et al., 2003; Dhond et al., 2007; Farahibozorg et al., 2022) as well as in multivariate analyses on intracranial recordings (Chan et al., 2011; Rogers et al., 2021).

A recent study examined the activation dynamics in several brain areas within the semantic network by contrasting two different tasks that differed with respect to the depth of semantic processing: semantic decision (SD) and lexical decision (LD; Rahimi et al., 2022). The evoked responses indicated that different semantic task demands are associated with an early modulation of visual and attentional processes, followed by differences in the semantic information retrieval in the ATLs, and finally a modulation in control regions (PTC and IFG) involved in the extraction of task-relevant features for response selection. These results were corroborated by the functional connectivity analysis in this and another study that revealed connectivity between the ATLs and semantic control regions (Rahimi et al., 2022, 2023a).

The present study aimed to extend the evoked results of Rahimi et al. (2022) using a multivariate decoding approach in source space. In contrast to the previous study, we investigated both stimulus and task features. In addition, we evaluated two different decoding approaches: per-ROI and across-ROI. We used these methods to compare the spatiotemporal decodability of stimulus and task features of dynamic brain activation patterns. In particular, we asked (1) whether the patterns that carry stimulus and task information overlap in space and time, and to what extent this information is distributed versus localized, and (2) whether the task affects the amount of available stimulus-specific information within the semantic network.

## Materials and Methods

We used the dataset described in Farahibozorg (2018) and Rahimi et al. (2022), and most preprocessing steps are identical to the latter. Thus, we will here report a summary of the most relevant characteristics, but refer to the previous study for more detailed information. Previous analyses of this dataset did not employ decoding of task or stimulus features.

### Code accessibility

The code for the analysis is available at https://github.com/magna-fede/SourceSpaceDecoding_SDvsLD.

### Participants

We analyzed data from 18 participants (12 female). All of them were native English speakers and right-handed, with normal or corrected-to-normal vision, and reported no history of neurological disorders or dyslexia. The study was approved by the Cambridge Psychology Research Ethics Committee.

### Stimuli and procedure

A total of 250 uninflected words were used in the visual stimulation. Each word belonged to one of five different categories based on its semantic content: visual, auditory, hand-action, emotional, or neutral abstract words. Table 1 summarizes the psycholinguistic variables. For the stimulus feature decoding, we used only a subset of the stimuli. Specifically, we created a subset of the two abstract word categories (i.e., emotional and neutral categories), so that we ended up with four semantic categories with 50 stimuli each. This was done because the stimuli in the emotional and neutral categories contained 1.5 letters more on average, compared with the other three categories. To avoid a false semantic classification due to word length, we removed this confound by selecting 50 words across these two categories, resulting in a more generic abstract word category.

**Table 1.** Summary statistics [mean and (standard deviation)] for each of the (single-word) semantic categories presented in both the SD and LD task and for pseudowords presented only in the LD task

	Number of letters	Frequency	Orthographic neighborhood size	Bigram frequency	Trigram frequency
Action	5.02 (1.20)	19.67 (30.86)	5.44 (5.25)	17,368.95 (9,580.53)	1,690.77 (2,505.16)
Auditory	5.16 (1.22)	11.03 (17.25)	5.28 (5.24)	17,599.13 (9,181.36)	1,825.17 (2,426.59)
Visual	5.18 (1.48)	13.32 (17.58)	4.50 (5.44)	20,089.52 (11,698.66)	1,867.21 (3,314.68)
Abstract	5.28 (0.93)	23.64 (23.76)	3.26 (3.84)	19,495.50 (9,564.39)	1,597.65 (955.62)
Pseudowords	5.00 (1.00)	N/A	4.70 (4.50)	19,465.83 (10,435.78)	1,670.66 (2,029.80)

The study consisted of four blocks and the full set of word stimuli was presented in each of them. One block consisted of a LD task, where participants were presented with 250 words and an additional 250 pseudowords. They were asked to press a button with either their left index or ring finger to indicate whether the letter string represented a real, meaningful word or not. The other three blocks were all different instances of a SD task: Participants were presented with the same 250 words as for LD, plus 50 additional fillers and 30 target words. Participants had to press a button with their left middle finger whenever the current stimulus belonged to the specific target semantic category for that block. The three target semantic categories were as follows: (1) “food that contains milk, flour, or egg,” (2) “noncitrus fruits,” and (3) “something edible with a distinctive odor.” The target categories were unrelated to the word categories of the stimuli described above. The order of presentation of the SD blocks was randomized; half of the participants performed the LD task first, and half of them did it after the SD blocks.

For the SD blocks, only nontarget trials were included in the analyses, that is, trials that did not require a button press response. For the LD task, participants responded with a button press to both real words and pseudowords since a two-alternative forced choice is the standard procedure in LD tasks. We did not consider the details of response execution at the end of each trial as a serious confound for our EEG/MEG results in earlier latency ranges [see Rahimi et al. (2022) for details]. These potential confounds are present only when classifying tasks in SD versus LD blocks, but not when considering the classification of different SD blocks or when performing single-word semantic classification.

### Data acquisition and source estimation

Simultaneous EEG and MEG data were concurrently recorded to maximize spatial resolution (Molins et al., 2008). The sampling rate during data acquisition was 1,000 Hz, and an online bandpass filter of 0.03–330 Hz was applied. Signal Space Separation with its spatiotemporal extension as implemented in the Neuromag Maxwell-Filter software was applied to the raw MEG data. Raw data were visually inspected for each participant, and bad EEG channels were marked and linearly interpolated. Data was then bandpass filtered between 0.1 and 45 Hz. The FastICA algorithm was applied to the filtered data to remove eye movement and heartbeat artifacts. We used L2-minimum norm estimation (MNE) for source reconstruction (Hämäläinen and Ilmoniemi, 1994; Hauk, 2004). Three-layer boundary element forward models were constructed using structural MRI scans.

### ROIs

**Figure 1.**
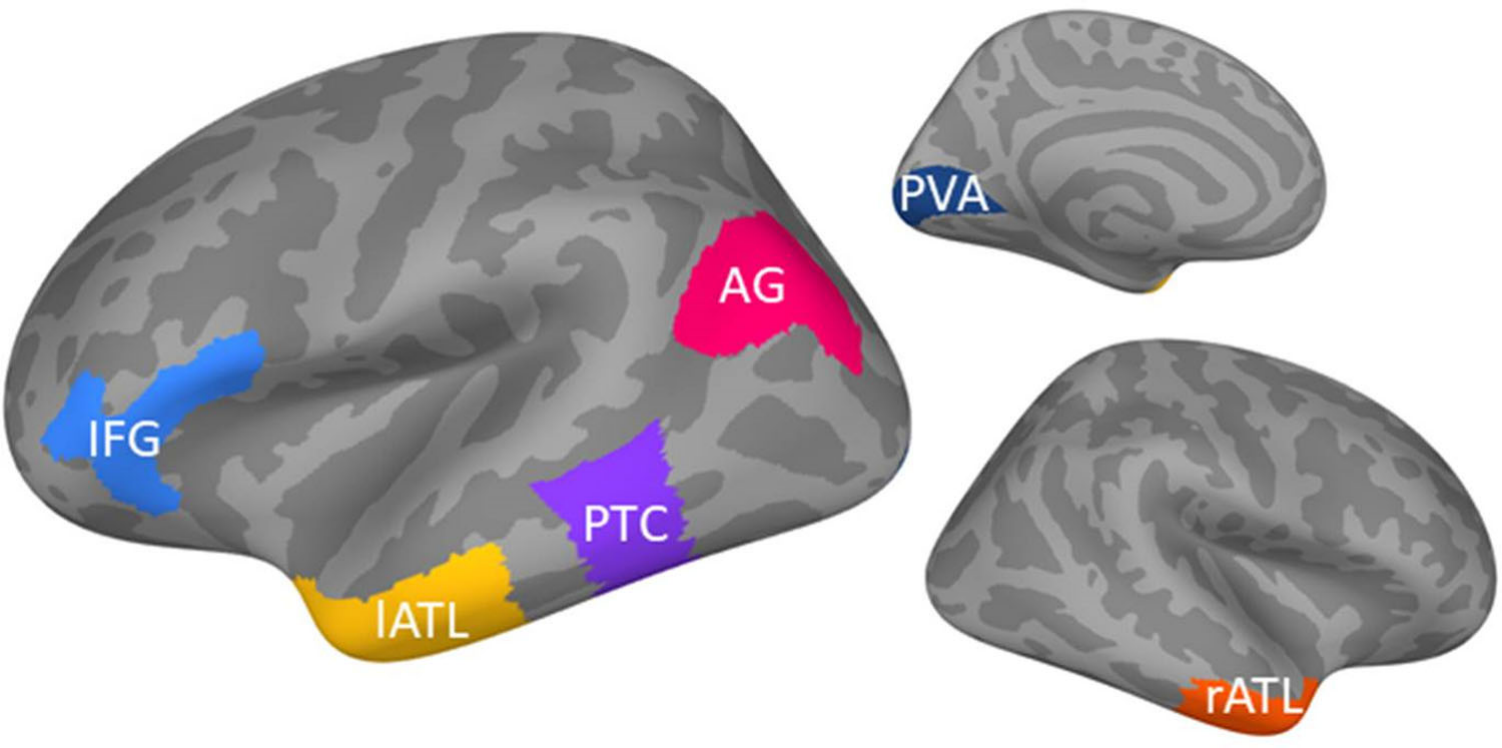
ROIs used in this study [based on Rahimi et al. (2022)].

We focused our analyses on six ROIs that were defined using the anatomical masks provided by the Human Connectome Project parcellation (Glasser et al., 2016). We adhered to the selection of Rahimi et al. (2022), who examined the evoked responses and functional connectivity analysis of these same ROIs (Fig. 1). These areas were chosen due to their putative role in the semantic brain network, as derived from neuroimaging fMRI studies. ATLs have been proposed to be multimodal semantic hub regions (Lambon Ralph et al., 2017). The IFG and PTC have been described as semantic control regions required for the appropriate task- or context-relevant processing of a word stimulus (Jackson, 2021). Angular gyrus (AG) has been suggested as an additional hub region or convergence zone (Binder, 2016). Finally, we included primary visual areas (PVA) to test potential task effects on early perceptual processing.

### Preprocessing

**Figure 2.**
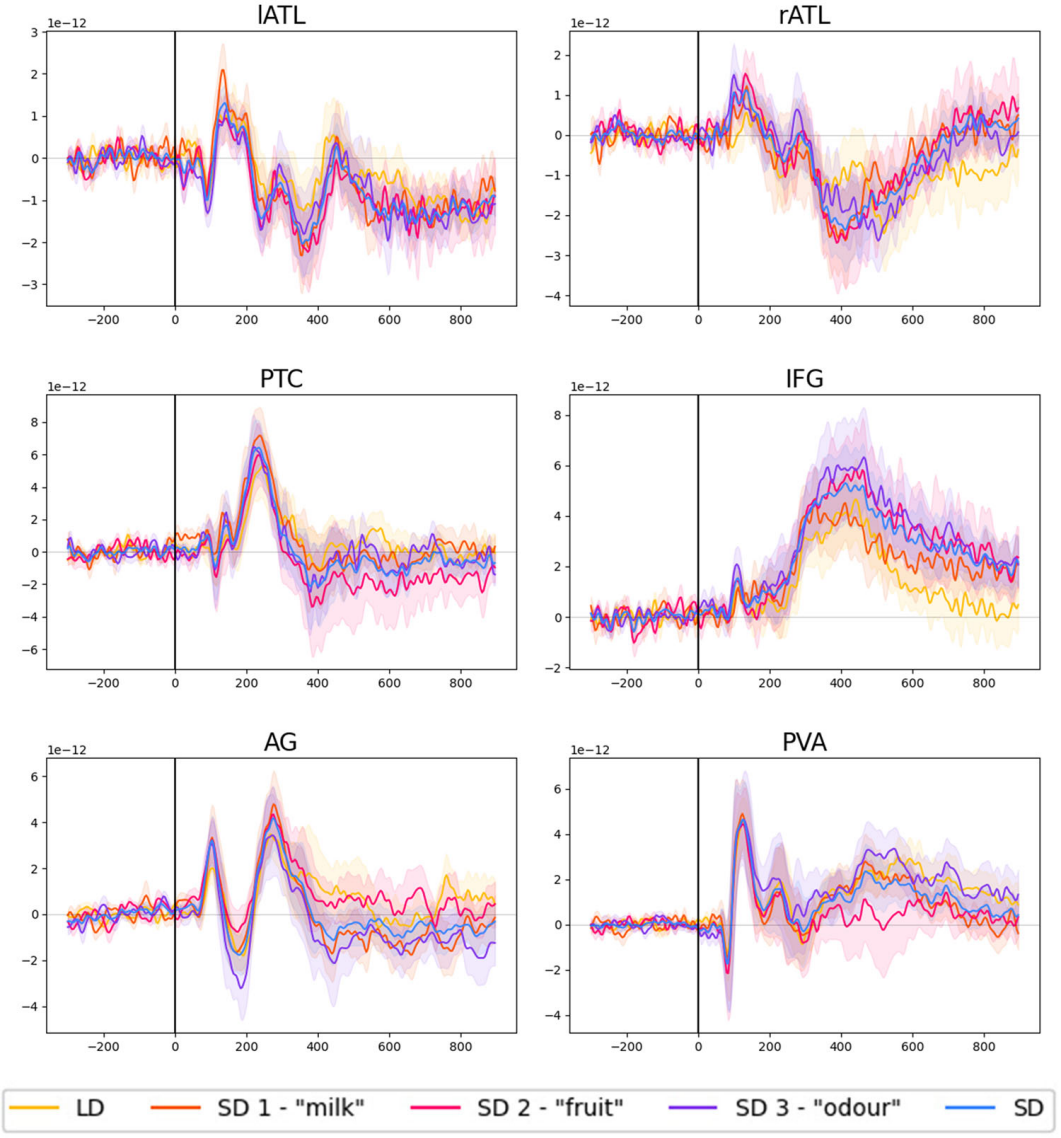
Evoked responses for several ROIs in source space for each task and SD tasks averaged together. The shadowed areas report standard error of the mean (LD, lexical decision; SD, semantic decision; “milk,” “fruit,” and “odor” decisions refer to single SD blocks).

The preprocessing and decoding analyses are based on tutorials and examples available on MNE-Python (Gramfort et al., 2013) and on the tutorial provided by Grootswagers et al. (2017). We estimated brain responses in source space for each participant and for each trial. We sampled our data in epochs that started from 300 ms before the stimulus onset until 900 ms poststimulus. Data was downsampled to 250 Hz. For the decoding analysis, we intended to keep as much information in our activation patterns as possible and therefore used signed source estimates, that is, rather than taking the (only positive-valued) intensities of dipole sources at each vertex, we kept their directions of current flow. We present the grand-averaged time courses of brain activation for each ROI and for different tasks in Figure 2 (using the “mean-flip” option to account for the variability of source polarities). The figures show that we obtained clear evoked responses in all ROIs and conditions.

The preprocessing consisted of averaging three trials together for each class, in order to reduce noise in the data and improve decoding accuracy (Isik et al., 2014). We then *z*-score–normalized each of the resulting epochs. The models fitted were always logistic regression models, using a five-fold cross-validation procedure. We calculated accuracy as the average receiver operator characteristic (ROC) area under the curve (AUC) on the test data across the five folds.

### Decoding analysis

We first decoded the task in two different ways: For the LD versus SD classification, we classified trials based on whether they were part of a LD or SD block (i.e., we only focused on real words, excluding pseudowords from the analysis). For the SD versus SD classification, we used multinomial logistic regression to predict to which of the three SD blocks a trial belonged, which in practical terms meant that we classified which question a participant was answering. Then, we decoded single-word semantic categories, separately for LD and SD blocks and compared the accuracies. For each classification analysis, we compared two different decoding approaches: (1) individual ROIs, wherein we estimated decoding accuracy in each ROI separately; (2) combined ROIs, wherein we fitted a model that used all vertices across all ROIs and then determined the contribution of each ROI by looking at the root mean square of the back-projected weights of the model within ROIs.

#### Individual-ROI accuracy

In the LD-SD classification, we separately classified each of the LD-SD combinations using a binary logistic regression model, and accuracy was calculated as the average across them. In the SD-SD and stimulus feature classification, we fitted multinomial logistic regression models and calculated accuracies as one-versus-rest ROC AUC (this ensured that the chance level was 0.5 for all tasks). For the semantic category analysis, we fitted a multiclass logistic regression model. For SD blocks, we estimated accuracy both when concatenating the three blocks in one model (in order to have more trials and therefore a more reliable estimate of regions and time points where stimulus features were decodable) as well as separately per block (to have a fair comparison in terms of noise level with the LD block with a comparable number of trials).

We performed statistical significance analyses to test differences in accuracy between different classification tasks, specifically, we tested (1) whether task decoding accuracy differed between LD-SD and SD-SD; (2) whether we could reliably decode word category classification above chance; and (3) whether stimulus feature decoding accuracy differed between LD and SD tasks. We used a cluster-based permutation test over time to correct for multiple comparisons (Maris and Oostenveld, 2007).

#### Combined-ROI patterns

In this analysis, we decoded patterns combined across ROIs and then determined how much each ROI contributes to the corresponding classifier. For this purpose, we back-projected the classifier weights into the corresponding activation patterns across ROIs as explained by Haufe et al. (2014). This back-projection is required because the classifier weights may reflect both signal and noise, while the back-projected patterns reflect brain activity predicted by the estimated model.

In general, we followed the same steps as in the individual-ROI procedure described in the previous section and subsequently extracted the back-projected weights of each feature in the model, which we will refer to as activation patterns or just patterns from now on.

For each classification task, we will report the root-mean-squared (RMS) activation patterns for each ROI, averaged across participants. As each model had a set of weights specific to each class, we will first calculate an ROI's RMS of each class-specific activation pattern—for example, in the semantic classification, ROI-specific hand-class patterns (and analogously for visual, auditory, and abstract classes). Then, by averaging across classes, we obtain the average activation pattern time course of each ROI, for each participant. The RMS pattern is a measure of the relative contribution of a certain ROI to the classification: Each vertex's value reflects the relative contribution of that specific vertex to the signal used by the classifier, that is, vertices that are close to 0 are relatively less informative, whereas larger absolute values indicate that the vertex contains more information. By considering the RMS instead of the average, we avoid that patterns of vertices within the same ROI with different polarities will cancel each other.

## Results

**Figure 3.**
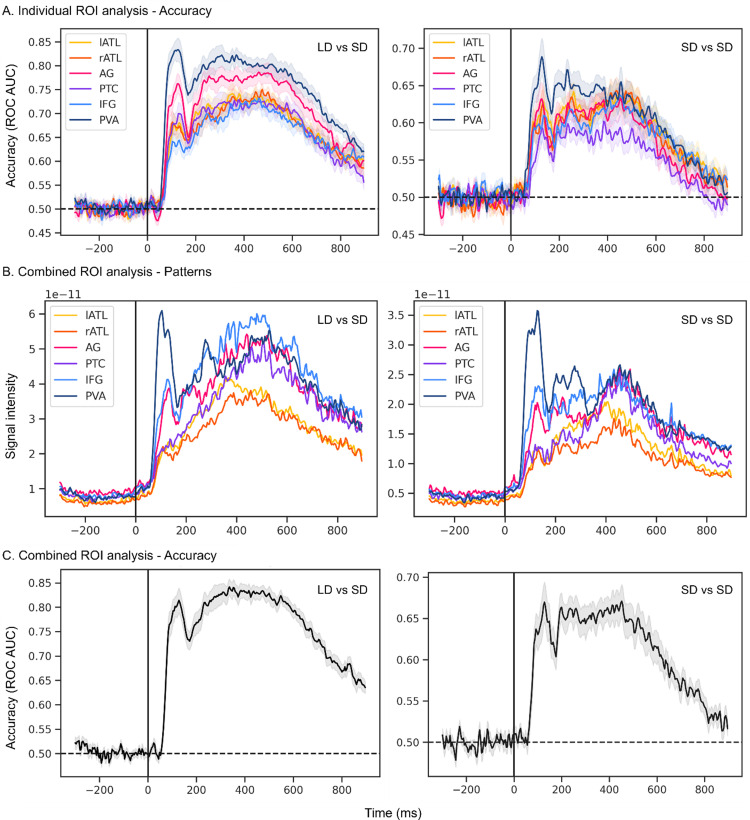
Task classification performance. For all plots, the shadowed area represents the standard error of the mean (across participants). ***A***, Individual-ROI task classification accuracy (ROC AUC) when decoding LD from SD (left) and when decoding SD tasks (right). ***B***, Root mean square of the activation patterns within each ROI, when decoding LD from SD (left) and when decoding SD tasks (right). ***C***, Combined-ROI task classification accuracy (ROC AUC) when decoding LD from SD (left) and when decoding SD tasks (right).

**Figure 4.**
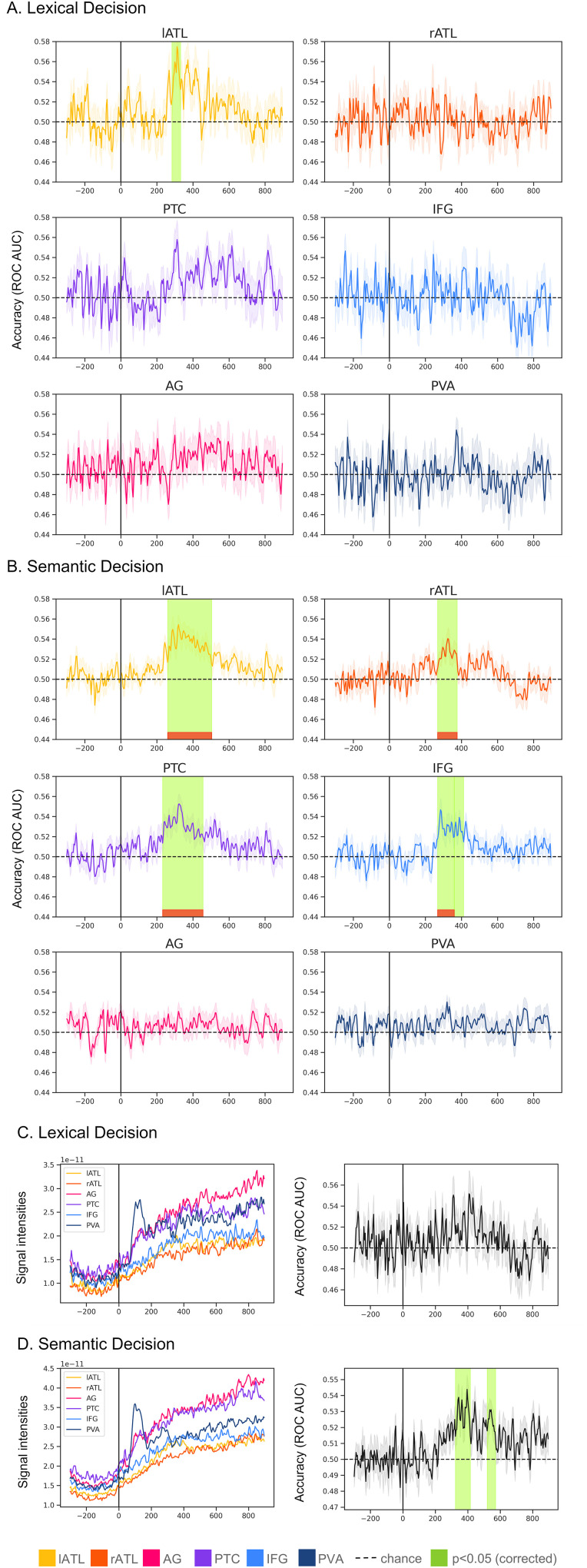
Single-word semantic category classification performance. For all plots, the shadowed area represents the standard error of the mean (across participants). The green color highlights the times when the cluster-based permutation correction test revealed a significant cluster (*α *= 0.05) where accuracy was above 0.5, and the red the clusters that are still significant after Bonferroni’s correction (across ROIs). ***A***, Individual-ROI task classification accuracy (ROC AUC) when decoding semantic features in LD block. ***B***, Individual-ROI task classification accuracy (ROC AUC) when decoding semantic features in SD blocks. ***C***, Combined-ROI root mean square of the activation patterns within each ROI (left) and classification accuracy (ROC AUC; right), when decoding semantic features in LD block when decoding LD. ***D***, Combined-ROI root mean square of the activation patterns within each ROI (left) and classification accuracy (ROC AUC; right), when decoding semantic features in SD block.

In the following, we will present results for different task classifications (LD-SD and SD-SD; Fig. 3) and semantic word category classification (Fig. 4). For each part, we will first present the results for individual-ROI classification followed by combined-ROI classification.

### Task classification

Figure 3 shows classification accuracy results for the task comparisons (left, LD-SD; right, SD-SD) over time for different ROIs in source space, separately for the individual-ROI approach (A) and the combined-ROI approach (B and C).

Both task classifications show similar results, in so far as the task was decodable from before 100 ms after stimulus presentation and then for most of the epoch, both in the individual- and combined-ROI approach. Detailed information is presented below.

#### Individual-ROI accuracy

In LD-SD, accuracy is at a chance level in the baseline period (before stimulus presentation), but it sharply increases after approximately 50 ms poststimulus reaching accuracies of over 0.7 in all examined ROIs at around 100 ms. Accuracy plateaus for a large part of the epoch and is followed by a slow decline. The SD-SD classification shows very similar results, although accuracy is generally lower, peaking below 0.7. Cluster-based permutation testing (not shown in figures) confirms this difference with LD-SD accuracy being significantly higher than SD-SD in all ROIs from around 70 ms poststimulus presentation to the end of the epoch (apart from IFG, where the difference starts at approximately 152 ms).

#### Combined-ROI patterns

When combining all ROIs in one model, the accuracy profile is very similar to that in the individual-ROI approach, that is, it is characterized by a sharp increase soon after stimulus onset and a plateau afterward (Fig. 3*C*). However, while the shape of accuracy time courses was very similar across ROIs in the previous analysis, we observed larger differences among ROIs when examining the RMS of activation patterns within ROIs (Fig. 3*B*). For example, PVA's (dark blue line) contribution to task classification (both LD-SD and SD-SD) peaks ∼100 ms. AG's and IFG's time courses (pink and light blue, respectively) are similar, with a smaller peak than PVA ∼100 ms but similar and even larger values after 250 ms. In contrast, the temporal lobes (lATL and rATL, yellow and orange, respectively, and PTC, purple) show similar time courses without a distinct early peak. Their activations slowly increase until they reach their peak ∼400 ms. Also in this case, cluster-based permutation testing (data not shown) showed that decoding accuracy was higher in LD-SD and SD-SD from ∼64 ms poststimulus onset.

Thus, while the individual-ROI analyses revealed that all ROIs carry information about the tasks, the combined-ROI provides more information about the time courses of information, in particular about their differential contributions at early (∼100 ms) versus later (>250 ms) latencies.

### Single-word semantic features classification

In the semantic category classification, for each stimulus, we decoded the semantic category it belonged to (out of 4 alternatives), separately for LD and SD blocks. Figure 4, *A* and *B*, shows the results for the individual-ROI stimulus feature decoding. Figure 4, *C* and *D*, shows the results for the combined-ROI pattern and accuracy. In general, we observed that stimulus features were decodable above chance level when participants were engaged in a SD task, but less so in LD.

#### Individual-ROI accuracy

Figure 4*A* revealed that in LD blocks only lATL showed significant decoding for word category and only in one short time window ∼300 ms. In contrast, for SD we obtained significant decoding in several regions (left and right ATL, PTC, and IFG). SD decoding survived Bonferroni’s correction across ROIs. For each ROI, we report the significant cluster(s) and their approximate latencies in Table 2. In SD, we observed above-chance decoding accuracy in left and right ATL, PTC, and IFG, but not in PVA and AG. PTC successfully classifies single-word semantic category between 236 and 452 ms poststimulus. This is followed by left and right ATL, respectively, between 264–500 and 272–372 ms. IFG was above chance approximately at the same time, between 282–356 and 364–408 ms (but the second cluster did not survive Bonferroni’s correction). As the SD model was trained on trials from three blocks, the table reports also the results of cluster-based permutation tests for each SD block separately, to show results from a comparable noise level to the single LD block. Decoding accuracy in single SD blocks was above chance in more ROI–latency combinations compared with the LD block (apart from the odor task).

**Table 2.** Approximate timings when a single-word semantic category is decodable in each ROI

	lATL	rATL	AG	PTC	IFG	PVA
LD	288–328 ms	-	-	-	-	-
SD	264–500 ms	272–372 ms	-	236–452 ms	272–356 ms 364–408 ms	-
Bonferroni corrected	264–500 ms	272–372 ms	-	236–452 ms	272–356 ms	-
SD 1—“milk”	-	316–372 ms	716–760 ms	-	588–632 ms	-
SD 2—“fruit”	348–404 ms	296–360 ms 544–592 ms	428–476 ms	316–408 ms 440–492 ms 788–844 ms	-	320–376 ms
SD 3—“odor”	-	-	-	-	248–292 ms	-

SD refers to the information when fitting a model to all SD blocks concatenated, whereas the effects for models fitted to individual SD blocks [i.e., (1) “milk,” (2) “fruit,” and (3) “odor”] are reported in the last three rows of the table.

**Figure 5.**
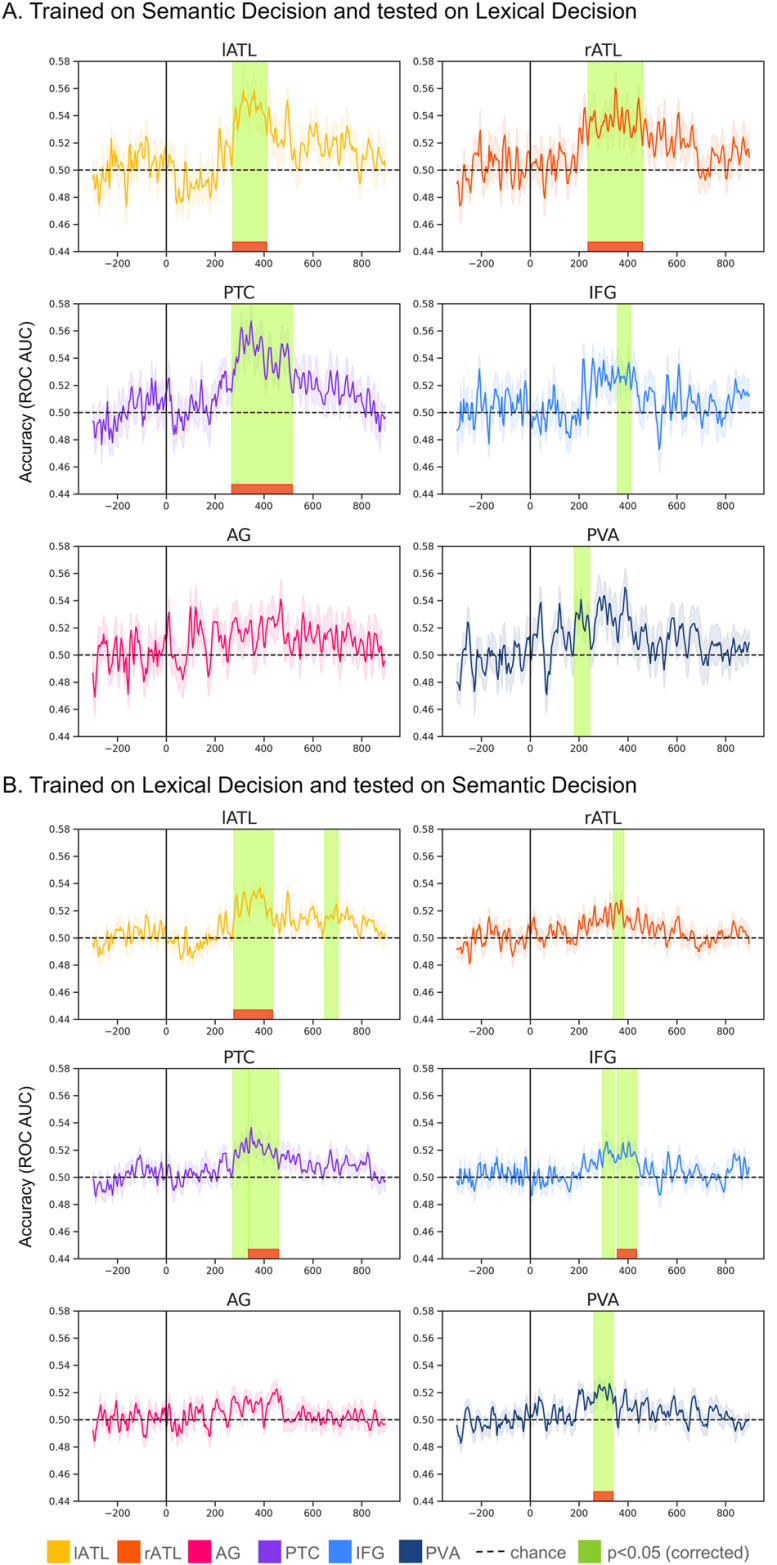
Cross-task semantic category decodability. For all plots, the shadowed area represents the standard error of the mean (across participants). Time points highlighted in green are times when the cluster-based permutation correction test revealed a significant cluster (*α* = 0.05). In red, are clusters that are still significant after Bonferroni’s correction (across ROIs). ***A***, Individual-ROI cross-decoding when the model was trained on SD trials and tested on LD trials. ***B***, Individual-ROI cross-decoding when the model was trained on LD trials and tested on SD trials.

We then statistically tested whether the decoding accuracy was significantly higher in SD than in LD considering that word category was decodable only in lATL in the LD block, in contrast to all the semantic network in SD. However, no ROI showed a significant effect. To further understand whether the task affected the stimulus category decoding, we computed cross-task decodability. This analysis was included to determine whether the representation of specific semantic information was consistent across tasks (i.e., successful cross-task decoding indicates that multivariate patterns of a certain word category are represented similarly irrespective of tasks). We trained a model on SD trials and tested stimulus features decodability of the same model in LD trials, and vice versa. The results are reported in Figure 5. When the model was trained on SD and tested on LD trials, we observed above-chance accuracy in all semantic areas: lATL between 276 and 408 ms, rATL between 240 and 456 ms, PTC between 272 and 512 ms, IFG between 360 and 408 ms, and PVA between 184 and 240 ms (where the last two regions did not survive Bonferroni’s correction across ROIs). We observed similar results in models trained on LD and tested on SD (although LD trials were significantly less so we will not interpret the results for each ROI).

**Figure 6.**
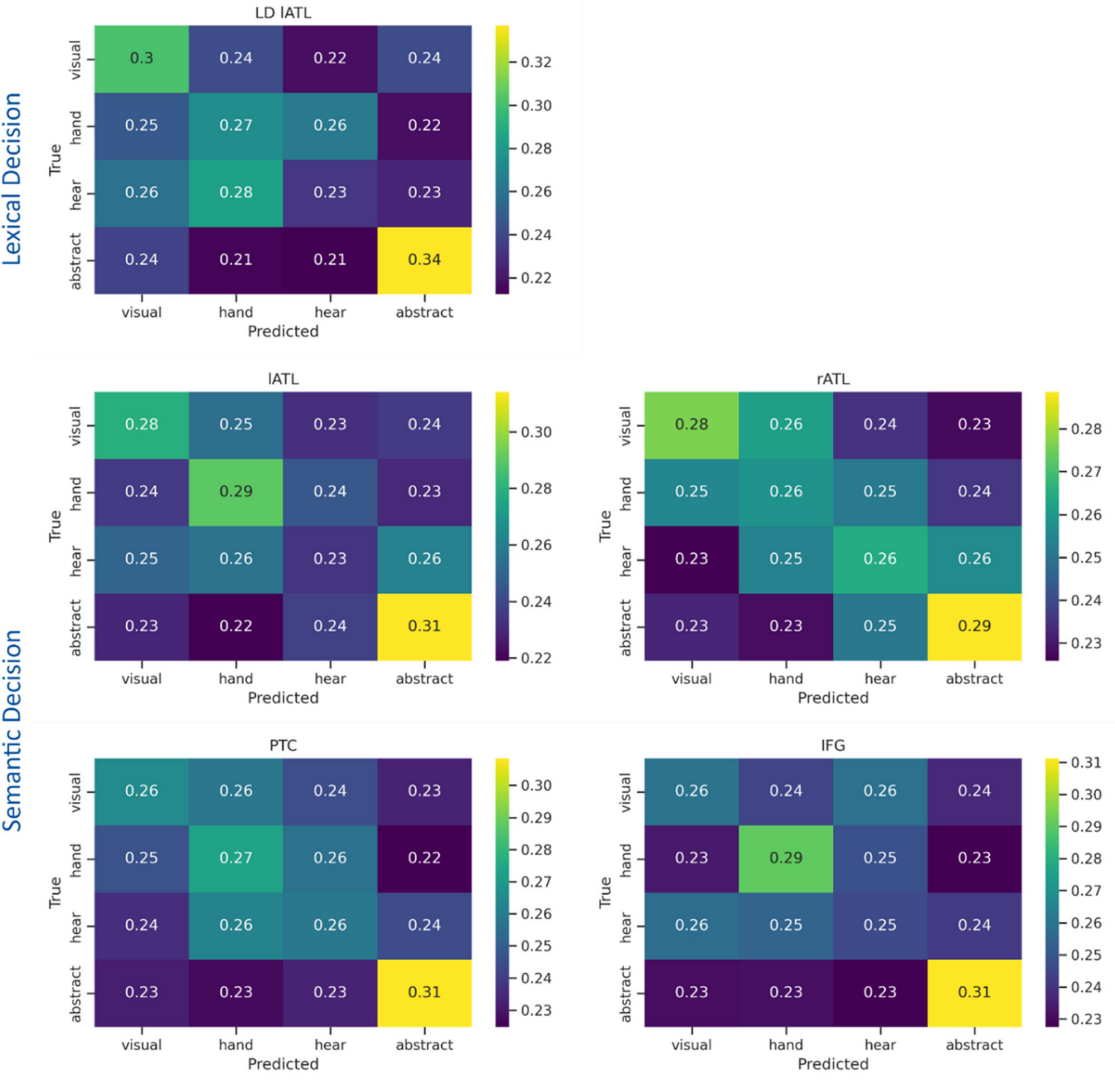
Confusion Matrices for the decoding of semantic word categories. Each cell represents the temporally averaged probability of a predicted word category, given the true word category, separately for each of the significant temporal clusters found in the semantic category classification. Smaller values indicate lower probabilities. In all cells, abstract words were the most accurately predicted category, compared with the other (concrete) words.

As a final step, we wanted to determine whether the decoding performance was driven by any particular semantic word categories, so we examined the confusion matrices of each significant temporal cluster. To obtain a confusion matrix for each cluster, we obtained the model's predicted class for each time point, separately for each participant. We then fitted a (normalized) confusion matrix for each point and considered the average values across time for each cluster. Figure 6 shows the confusion matrices for all the temporal clusters that were significantly above chance across participants in the semantic category classification, for each task/ROI separately. The confusion matrices indicate that abstract words are the most differentiable (i.e., relatively higher accuracy) from the other classes, which were all concrete.

#### Combined-ROI patterns

Similar to the individual-ROI case, accuracy was reliably above chance only in the SD but not LD task. In SD, one cluster extended between 328 and 412 ms and a second cluster approximately between 524 and 568 ms. However, also in this case, statistical analysis revealed no difference between SD and LD models. Decoding accuracy for individual ROIs as well as for combined ROIs was quite low (below 0.55) compared with our results in Figure 3. As a result, the activation patterns for the combined-ROI analysis appeared to be less informative in this case. For both LD and SD, these patterns showed an almost linear, drift-like increase over time following the baseline period, with a small peak at ∼100 ms for PVA. This confirms the assertion of Haufe et al. (2014) that classifier weights and activation patterns are less interpretable when decoding accuracies are low.

## Discussion

Our study had two main goals: First, we wanted to characterize the spatiotemporal dynamics within the semantic brain network using multivariate information decoding, and second, we compared two different approaches to source space decoding, based on individual-ROI and combined-ROI data.

Logistic regression models performed very well at classifying tasks and showed high accuracy in decoding LD versus SD trials in all ROIs from before 100 ms. This is consistent with previous studies that reported the effects of tasks on visual word processing from early latencies (∼100 ms, Chen et al., 2015) and the evoked and functional connectivity results obtained from the same data (Rahimi et al., 2022). However, these effects between LD and SD may not be surprising, since these tasks differ in several ways, such as the semantic depth of processing, task difficulty, and attentional demands (as reflected in response times), and stimuli were presented in different blocks. More interestingly, the classifier could distinguish with high accuracy between different SD blocks, which reflected more subtle task demands as they only differed with respect to the semantic target category for the response. In all task classifications, all ROIs showed an early increase in decoding accuracy from ∼50 ms, which was followed by a plateau, and a slow decline toward the end of the epoch. This plateau indicates that for a sustained period, the whole semantic network carries information about the task that is being performed. Importantly, decoding performance was at chance in the baseline period, indicating that our results are not confounded by the fact that tasks were performed in different experimental blocks.

One previous study examined the conjoint effects of task and stimulus in a multivariate MEG analysis (Toneva et al., 2020). The task consisted of answering different questions regarding the semantic properties of each stimulus, analogous to the SD task in our paradigm. Thanks to the great range of questions, they were able to create vector representations of both the stimuli and task, based either on Bidirectional Encoder Representations from Transformers (BERT, an open-source large language model) or ratings along a number of dimensions collected separately. The authors found that including task information improved the prediction of MEG recordings only very early between 50 and 75 ms and between 475 and 550 ms after stimulus onset. A model of the questions’ semantics performed better than chance around the latter latency range. The results are quite different compared with our results that showed decoding of tasks throughout most of the epoch, even within semantic tasks (SD vs SD). The first important difference compared with our study is that they only considered sensor space. Arguably, focusing on hypothesis-driven ROIs can lead to better decoding performance, by fitting a model on data where different activation patterns are expected. Another important difference between the two studies is in the object of the prediction: In our work, the classifier was trained to predict tasks based on neural activity, but in Toneva et al., it predicts neural activity based on task (and stimulus). Importantly, encoding and decoding models support different types of inferences: While Toneva et al.’s results showed that task affects the semantic processing of a word only in the final stages, our results are limited in showing that task information is present across the brain throughout the epoch. Another important difference is that Toneva et al. focused on the semantic representation of tasks, in the form of embeddings. Arguably, tasks are not just “semantic representations” as captured by vectorial representations but also have an active processing component, which is poorly captured by static vectorial semantics. This might explain why Toneva et al. observe a relatively short-lived influence compared with our results, as in multivariate pattern recognition everything that separates two classes is taken into consideration, but in an encoding model, the properties must be explicitly modeled (and in this case only the semantics is modelled).

Also, we were able to decode single-word semantic category across the whole semantic network (left and right anterior temporal lobe, left PTC, and left IFG) in the SD task and in the left anterior temporal lobe in the LD task. This indicates that semantic information is represented across all the semantic network, at least when performing a semantically engaging task. However, statistical tests revealed no significant differences between the two tasks in the direct comparison of any ROI. Thus, word category information may be present across the semantic network in both tasks but only reach significance in the SD task. This was confirmed by our additional cross-task decoding analysis: When training the classifier on SD and testing on LD trials, and vice versa, we could successfully decode stimulus features to a similar degree as in individual tasks. Thus, while a previous study revealed significant differences between tasks in evoked activity and functional connectivity (Rahimi et al., 2022), our current decoding results show that when it comes to semantic feature representations, there is a higher degree of similarity across tasks. Altogether, this supports the view that early visual word recognition processes are flexible, that is, they are modulated by task demands, but semantic information is processed even if not explicitly required by the task (Evans et al., 2012; Chen et al., 2015).

Only the AG and primary visual cortex did not produce above-chance decoding accuracy for semantic word category, neither in LD nor in SD blocks. This confirms previous results that early brain activity in the temporal lobes, but not in the AG, reflects semantic word features (Farahibozorg et al., 2022). From a CSC perspective, it is argued that the AG does not support semantic representation per se but is involved in more spatiotemporally extended semantic processing (Humphreys et al., 2021). This is supported by previous MEG analyses that used DCM (i.e., dynamic causal modeling) connectivity analysis (Farahibozorg et al., 2022). The authors showed that while in early time windows, the ATL acted as a hub (0–250 ms), for an extended time window (0–500 ms), the AG acted as a hub. However, as the AG is not modulated by semantic properties, its role is interpreted as a bridge from the semantic system to other brain systems involved in memory and higher cognitive functions. Importantly, this is in contrast with results from a number of functional MRI studies, which found reliable involvement of the AG in semantic processing (Binder et al., 2009; Fernandino et al., 2022). Source space estimation of the AG is unlikely the cause of this discrepancy, as the cortical morphology and the position of the AG allow for good estimation when using the most commonly available inversion technique (i.e., deep sources being, in general, more difficult to reliably estimate). The absence of PVA effects in the category decoding analysis indicates that perceptual properties of the stimuli were not influencing the classification performance, as our stimuli were well matched for several relevant psycholinguistic variables. Thus, while for pictorial stimuli semantic category is often confounded by visual stimulus features (e.g., natural concepts being more “curvy” and artifacts being more “edgy”), our word recognition results are most likely due to semantic stimulus features.

One issue with whole-brain decoding in source space is that the number of features can be quite large, especially compared with sensor space. While one can apply either feature selection (such as ANOVA *f*-test) or dimensionality reduction (such as PCA), we decided to focus on brain ROIs determined a priori, based on previous studies (Rahimi et al, 2022; Farahibozorg et al., 2022) and derived from the CSC framework (Jackson, 2021; Lambon Ralph et al., 2017). However, it is possible that other regions contain semantic information (Huth et al., 2016; Fernandino et al., 2022), and future studies may decide to use a searchlight approach in order to uncover other brain areas that contain semantic information.

Finally, we tested whether and how different semantic features affected the decoding performance in different regions. We addressed this question by computing confusion matrices for the decoding of different word categories against each other. This revealed that concrete word categories were more likely to be confused with each other (mostly indistinguishable) and that abstract words were driving the decoding performance across all regions that showed above-chance performance (i.e., all the semantic network in the SD task). This confirms that information about a word's concreteness is represented in the activation patterns within the semantic brain network from early latencies, as demonstrated in previous studies at a univariate level (e.g., Farahibozorg et al., 2022). Also, this indicates that multivariate brain patterns reflect semantic similarities (i.e., more similar concepts have similar representations) and parallel fMRI results (Kriegeskorte et al., 2008).

Overall, our results show that MEG data, and especially source space data, can uncover the dynamic processes that support semantic cognition. Indeed, a recent study by Bezsudnova et al. (2023) showed cross-modality decoding of images and text. This suggests that information regarding conceptual representations is not only stable across tasks (as found in our study), but also across modalities, although sensitive to time constraints determined by previous computation stages (e.g., visual processing). While the majority of neural semantic decoding studies still focus on fMRI data (Rybář and Daly, 2022), MEG data contains rich information that can be exploited with multivariate analyses both from a temporal point of view and, when performed in source space, from a spatial point of view (Kietzmann et al., 2019). Future studies should consider using deep neural networks to perform decoding of semantic information in more naturalistic tasks [Défossez et al., 2023; as done in fMRI (Tang et al., 2023) or ECoG (Goldstein et al., 2023)] to gather more fine-grained information which could give us greater insights into language processing in different brain regions.

A methodological objective of our study was to compare two approaches to source space decoding. We looked at both task classification and stimulus classification using decoding per ROI and decoding across ROIs. While we found reliable task decoding using both approaches, for the semantic category decoding, only the individual-ROI approach yielded reliable results. This suggests that when the effect of interest is distributed across brain regions, then the across-ROI approach is more informative, but when the effect of interest is distributed more sparsely in space, that is, if it is present only in regions, then the per-ROI approach is more sensitive.

Decoding performance was high both for general (LD-SD) and more subtle (SD-SD) tasks, across all our ROIs. Although in both cases accuracy varied across ROIs (e.g., relatively higher in PVA and AG compared with semantic ROIs in the LD-SD task), it is not straightforward to interpret this as evidence of different degrees of information contained in each ROI, as different algorithms and parameters will likely influence the accuracy score. For example, this difference could just reflect the “visibility” of a region by the EEG/MEG sensors. Hence, the individual-ROI approach was not very informative concerning the time course of the effects (contrary to the semantic category decoding), as all ROIs showed a similar time course (rapid increase in performance, followed by a plateau, and slow decline). However, more information was available when observing the activation patterns of the model that combined across-ROI information. For example, we observed that while the relative contribution of visual areas starts early, semantic regions take longer to reach their peak, consistent with univariate results of a posterior-to-anterior sweep of information, which has been observed in evoked responses (Marinkovic et al., 2003; Rahimi et al., 2022). Interestingly, we found no obvious difference between ROIs relevant for the LD-SD and SD-SD classifications.

The only region that showed significant decoding accuracy across all our analyses was the left ATL, with the caveat that the differences in stimulus decoding accuracy did not differ statistically between the lexical and semantic decision tasks. Nevertheless, we found involvement of those regions that have been put forward as the core semantic network (Lambon Ralph et al., 2017). More specifically, it has been suggested that IFG exerts semantic control function via PTC onto ATL (Jackson et al., 2021). Our results are consistent with this framework. Information regarding the semantic properties of a word seems to be spread across the core semantic network, but probably not outside (at least not in PVA and AG). This semantic information is at least partially stable across tasks. Future studies should investigate the information flow based on multivariate patterns as well as connectivity analyses in more detail. The amount of multivariate methods available for evaluating semantic computations from neuroimaging data is rapidly increasing [see Frisby et al. (2023) for a review]. Furthermore, methods that characterize brain connectivity based on multidimensional relationships, or pattern-to-pattern transformations, have recently become available (Basti et al., 2020; Rahimi et al., 2023a,b).

In conclusion, our results demonstrate that EEG/MEG source space activity contains rich information about stimulus and task features in written word recognition, which will be essential to unravel the complex dynamics in the semantic brain network.

## Synthesis

Reviewing Editor: Anne Keitel, University of Dundee Decisions are customarily a result of the Reviewing Editor and the peer reviewers coming together and discussing their recommendations until a consensus is reached. When revisions are invited, a fact-based synthesis statement explaining their decision and outlining what is needed to prepare a revision will be listed below. The following reviewer(s) agreed to reveal their identity: NONE.

The reviewers and editor assessed the manuscript overall positively. They agreed that the main issue was that the introduction and discussion are not sufficiently linked to previous research. In a revised version of the manuscript, please embed the current study more broadly in previous research and specifically highlight any potential novel contributions and differences with other studies. In addition, more methodological details are required to fully understand the experimental approach and analyses.

Please see below for the unabridged reviewer comments, which detail these issues further, as well as other issues. Please respond to each of the comments in a point-by-point manner.

*****

Reviewer #1 - Advances the Field (Required)

The results suggest that task effects engage a distributed semantic network, including bilateral anterior temporal lobes and control regions. In contrast, stimulus effects are more focal.

Reviewer #1 - Statistics

I found that the statistical analyses was correct and sound.

Reviewer #1 - Comments to the Authors (Required)

The study by Magnabosco & Hauk applied a decoding approach to disentangle task-related (comapring lexical decision (LD) and three semantic decision (SD) tasks), and stimulus-related effects in the semantic networks. The results suggest a that task effects engage a distributed semantic network, and stimulus effects are more focal. The results also highlight a joint role of bilateral anterior temporal lobes and control regions.

The methods are sound and the results robust. The study is clearly written. The only concern that I have is the narrow scope of the introduction and discussion, with limited references to the study in the field outside the authors' work. I have a few general comments below.

General comments

"These results inform current neuroscientific models of controlled semantic cognition" Please briefly clarify how, what the results tell us about current models we did not know already.

"While fMRI studies often focus on spatial information of decoding accuracies across different

cortical regions (e.g., Haxby et al., 2001; Huth et al., 2016)". Do the authors mean regions of interest or whole-brain decoding? Please clarify.

The introduction assumes a role in representation of the hub ATL, and a role in control of IFG and posterior temporal cortex. The AG is introduced in the method section as ad additional convergence zone. How does the AG fit the theoretical picture of the two representation and control dimensions?

In the result section, it is claimed that "AG does not support semantic representation per se, but is involved in more spatiotemporally extended semantic processing (Humphreys et al., 2021)." How does this result relate to the work by Fernandino et al., 2022-2021 etc?

What I feel is missing is a paragraph comparing the outcome of the current study to earlier literature, stressing what the new results add to the field. Please expand the MEG literature.

Reviewer #2- Advances the Field (Required)

It seems that current work might not offer a significant novelty compared to previous research studies, as these works already evaluated regional-level performance in naturalistic stimuli settings (Goldstein et al. 2022, Toneva et al. 2021).

Reviewer #2- Comments to the Authors (Required)

Prior studies on brain encoding and decoding have examined situations where participants were engaged in tasks related to either straightforward concepts (lexical) or stimulus presentations accompanied by relevant questions (semantic tasks). The main objective of these studies was to evaluate the predictive efficacy of diverse perceptual and semantic feature models for MEG data associated with concept words. These investigations also underscored the importance of the task question preceding the concept word, particularly in terms of understanding how the semantics of the task question influence subsequent processing and neural activity synchronized with the stimulus word. However, current EEG/MEG studies have predominantly focused on decoding accuracy over time in sensor space, rather than at the level of specific regions of interest (ROIs).

This paper delves into the temporal dynamics of the semantic brain network using EEG/MEG data to decode both task and stimulus information. The experimental findings reveal that the effects of decoding tasks remained consistent across various regions from 50-100 ms, regardless of differences in semantic tasks. In contrast, decoding the semantic word categories was constrained to specific regions between 250-500 ms. When comparing decoding approaches, traditional ROI-by-ROI decoding yielded better results for word-category decoding, while combined-ROI decoding using back-projected activation patterns offered more informative outcomes for task decoding. This underscores the distributed nature of the semantic network.

The paper contains the following key contributions:

1. The contributions of this work lie in its focus on ROI-level decoding for both lexical word and semantic decision processing.

2. Like prior research works, current study also examined both stimulus and task features concurrently. Furthermore, authors assessed two distinct decoding methods: one focusing on individual regions of interest (per-ROI) and the other spanning multiple regions (across-ROI).

3. Specifically, authors addressed two key questions: firstly, whether there is an overlap between the spatial and temporal patterns carrying stimulus and task information, indicating the degree of information distribution versus localization; and secondly, whether the task influences the quantity of stimulus-specific information accessible within the semantic network.

Main Review:

Weaknesses:

1. It seems that current work might not offer a significant novelty compared to previous research studies, as these works already evaluated regional-level performance in naturalistic stimuli settings (Goldstein et al. 2022, Toneva et al. 2021).

Goldstein et 2022, Correspondence between the layered structure of deep language models and temporal structure of natural language processing in the human brain, Nature 2022.

Toneva et al. 2021, Modeling Task Effects on Meaning Representation in the Brain via Zero-Shot MEG Prediction, NeurIPS-2021

2. While the authors mention two approaches, namely lexical decision and semantic decision tasks, it is unclear how they represented the task features. The lexical decision task is relatively straightforward, involving logistic regression to classify words based on MEG patterns. However, the commenter notes that the study lacks clarity regarding the representation of task features, which may impact the understanding of the experimental approach.

3. Authors showcase interesting results for both ROI-ROI and Combined ROI levels. However, it suggests that a more detailed analysis could provide deeper insights, emphasizing that a comprehensive exploration might offer more valuable information than what is already available in the existing literature.

4. Specifically, the authors could focus on decoding POS tag entities, dep tags, morphological features. This additional analysis could potentially offer a clearer picture of how these linguistic elements relate to the functioning of different brain regions.

Quality: The paper supports its claims with limited details. Although the paper is well-written and easy to follow. The authors missed some recent works that studied word and task-level information for MEG brain encoding.

Authors missed the following papers:

Goldstein et 2022, Correspondence between the layered structure of deep language models and temporal structure of natural language processing in the human brain, Nature 2022.

Toneva et al. 2021, Modeling Task Effects on Meaning Representation in the Brain via Zero-Shot MEG Prediction, NeurIPS-2021

Clarity: The information provided in the submission is not sufficient to reproduce the results.

Decision: Revision is required clear methodological details, motivation while comparing with previous works.
